# Atrial myxoma presenting with orthostatic hypotension in an 84-year-old Hispanic man: a case report

**DOI:** 10.1186/1752-1947-3-9328

**Published:** 2009-12-14

**Authors:** Ralph M Vicari, Enrique Polanco, Norberto Schechtmann, José O Santiago, Kautilya Shaurya, Michael Halstead, Danielle Marszal, Tamsin Grosskreutz, Shalini Thareja

**Affiliations:** 1 Mima Century Research, E. Sheridan Rd, Melbourne, FL 32901, USA; 2 Miller School of Medicine, NW 14th St, Miami, FL 33136, USA; 3 Tulane University, St Charles Avenue, New Orleans, LA 70118, USA; 4 University of Central Florida, Central Florida Blvd, Orlando, FL 32816, USA; 5 Florida Atlantic University, Glades Rd, Boca Raton, FL 33431, USA; 6 Columbia University, Haven Ave, New York, NY 10032, USA

## Abstract

**Introduction:**

Left atrial myxomas remain the most common benign primary cardiac tumors, and these cardiac growths can masquerade as mitral stenosis, infective endocarditis and collagen vascular disease. Atrial myxomas are found in approximately 14-20% of the population and can lead to embolization, intercardiac obstructions, conduction disturbances and lethal valve obstructions.

**Case presentation:**

An 84-year-old Hispanic man presented with complaints of dizziness upon standing, and with no prior history of heart murmurs, syncope, shortness of breath, or chest pain. Physical examination revealed evidence of orthostatic hypotension and a soft grade 1/6 systolic murmur at the left sternal border. A transthoracic echocardiogram revealed a large atrial myxoma occupying the majority of the left atrium, with the posterior border of the large atrial mass defined by eccentric mitral regurgitation identified during cardiac catheterization. Left atrial myxoma excision was performed, revealing a 7 × 6.5 × 4.5 cm atrial tumor attached to a 4 × 3 × 2 cm stalk of atrial septal tissue.

**Conclusion:**

This patient didn't present with the common symptoms associated with an atrial myxoma, which may include chest pain, dyspnea, orthopnea, peripheral embolism or syncope. Two-dimensional echocardiography provides substantial advantages in detecting intracardiac tumors. We recommend a two-dimensional echocardiogram in the workup of orthostatic hypotension of unknown etiology after the common causes such as autonomic disorders, dehydration, and vasodilative dysfunctions have been ruled out. By illustrating this correlation between orthostasis and an atrial myxoma, we hope to facilitate earlier identification of these intracardiac growths.

## Introduction

Although quite rare, left atrial myxomas account for 80% of all cardiac tumors. Diagnosis is often difficult due to the wide array of presenting symptoms. Atrial myxomas are associated with systemic embolization in 30 to 40% of cases [1]. These intracardiac growths may masquerade as mitral stenosis, infective endocarditis, and collagen vascular disease, which can further impede accurate diagnosis. The discriminatory marker for an atrial myxoma is often a tumor 'plop' heard upon auscultation at the apex of the heart.

We present the case of an 84-year-old man with a large atrial myxoma, who presented with complaints of positional dizziness and who was found to have a grade 1/6 systolic murmur, and significant orthostatic hypotension.

## Case presentation

An 84-year-old Hispanic man presented with complaints of dizziness upon standing, which was relieved by lying down. Physical examination revealed a drop in the patient's blood pressure from 124/80 mmHg supine to 99/70 mmHg one minute after standing. Pulse rate during the examination remained static. The patient had no prior history of heart murmurs, syncope, shortness of breath, or chest pain. Further physical examination revealed a soft grade 1/6 systolic murmur at the left sternal border, with no diastolic murmur present. There was no evidence of a tumor 'plop'.

**Figure 1:**
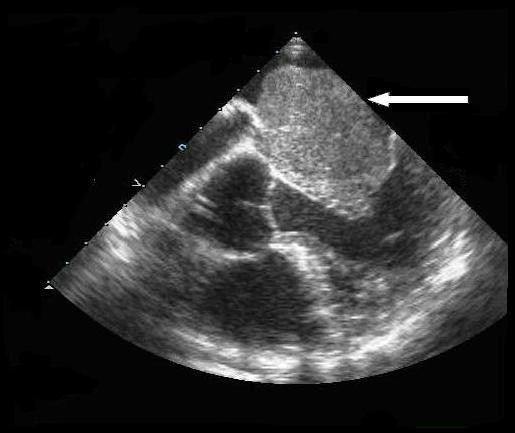
**Atrial myxoma: prolapsing through mitral valve**. Eleven days pre-operatively, the left atrium and left ventricle are visualized in this transesophageal echocardiogram.

A transthoracic echocardiogram was performed that revealed a large atrial myxoma occupying the majority of the left atrium. Cardiac catheterization showed eccentric mitral regurgitation, defining the posterior border of the large atrial mass. Transesophageal echocardiography, carried out at the time of surgery, revealed a large myxoma prolapsing through the mitral valve leaflets into the left ventricle (Figure 1).

**Figure 2:**
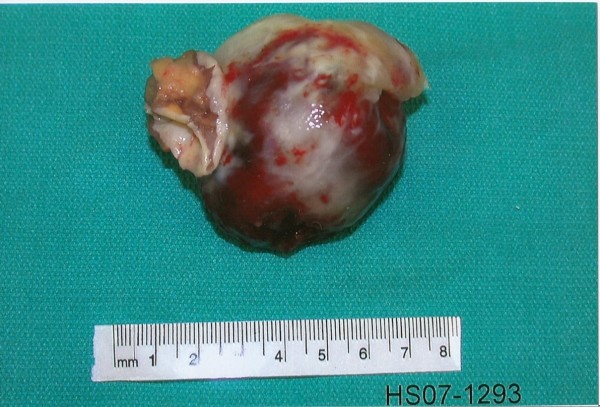
**Postoperative atrial myxoma**. The atrial tumor seen directly postoperatively was a mass of 7 × 6.5 × 4.5 cm attached to a 4 × 3 × 2 cm piece of atrial septal tissue.

A left atrial myxoma excision was performed, resulting in successful removal of the tumor. Pathological analysis of the atrial mass revealed it to be 7 × 6.5 × 4.5 cm attached to a 4 × 3 × 2 cm stalk of atrial septal tissue (Figure 2). Four weeks postoperatively, the patient stated that the original complaint of 'dizziness upon standing' had disappeared, with no evidence of orthostatic hypotension during a follow-up physical examination. A follow-up echocardiogram showed no evidence of atrial myxoma recurrence, and the mitral valve leaflets separated normally without regurgitation.

## Discussion

Our patient failed to present with the common symptoms associated with atrial myxoma including chest pain, dyspnea, orthopnea, peripheral embolism or syncope. Though cardiac myxomas are known to present with various non-specific clinical symptoms [2], orthostatic hypotension is not listed as a presenting symptom of atrial tumors in most textbooks of internal medicine or cardiology [3,4]. An extensive literature search revealed one case that reported orthostasis as a presenting symptom of a left atrial myxoma [5]. The patient in that case report had a principal complaint of dizziness upon standing, and orthostasis was observed with a blood pressure change from 90/50 mmHg supine to 64/40 mmHg standing. Upon echocardiographic investigation, a large atrial myxoma was found impeding inflow into the ventricular cavity upon standing. The myxoma was 3.5 cm in diameter and attached to the postero-inferior portion of the left atrial wall. This atrial tumor, smaller than the one we describe, brought on symptoms of orthostasis similar in severity to those that we observed in the patient in this case report. Upon removal of the myxoma in the Takemura case and in the case we describe, all clinical symptoms of orthostasis were relieved [5].

Orthostasis is relatively common in the elderly, being found in nearly 5-30% of the population with common causes including neurogenic dysfunction, autonomic failure, antihypertensive medications and intravascular volume depletion [6]. Since orthostatic hypotension is frequent in the elderly and there are numerous known causes for its occurrence, atrial tumors may be overlooked as the culprit for the manifestation. Since both cases discussed presented positional dizziness as the sole presenting symptom, we believe it is important to include atrial myxomas in the differential diagnosis of orthostasis.

## Conclusion

Two-dimensional echocardiography provides substantial advantages in detecting intracardiac tumors. A two-dimensional echocardiogram is recommended by the authors of this report in the workup of orthostatic hypotension of unknown etiology. Although atrial myxomas are usually benign or asymptomatic, there is the possibility of diastolic embolization [7], conduction alterations and disturbances, and lethal valve obstructions occurring [4]. Since surgical excision has been reported to alleviate symptoms associated with cardiac myxomas, early identification and removal is preferable. By illustrating this correlation between orthostasis and atrial myxomas, we hope to facilitate earlier identification of these intracardiac growths.

## Consent

Written informed consent was obtained from the patient for publication of this case report and any accompanying images. A copy of the written consent is available for review by the Editor-in-Chief of this journal.

## Competing interests

The authors declare that they have no competing interests.

## Authors' contributions

RV came up with original conception and design. RV, EP, NS, and JS scientifically reviewed and edited the study. KS, MH, DM, TG, and ST reviewed the medical literature, and were major contributors in writing the manuscript. KS, DM, and TG formatted the media. All authors read and approved the final manuscript.
